# Picture quiz

**Published:** 2018-11-09

**Figure:**
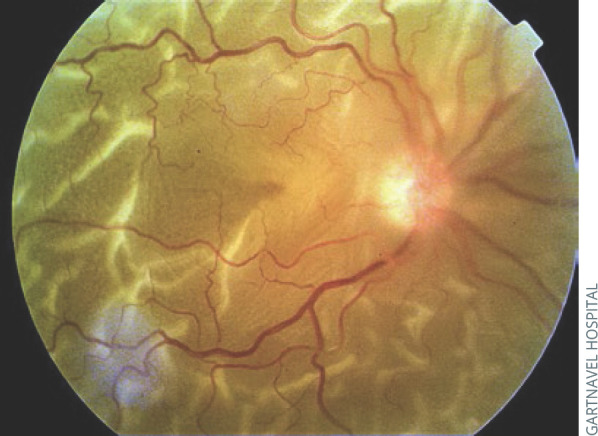


This is a fundus photo of a 60-year-old man, who had successful cataract surgery three years ago. His visual acuity is hand motions, and he complains of sudden, painless loss of vision in his right eye two weeks ago.

**Tick ALL that are TRUE**

**Question 1 What is the most likely diagnosis?**
  □ **a.** Optic atrophy
  □ **b.** Chronic open-angle glaucoma
  □ **c.** Posterior capsule opacification
  □ **d.** Retinal detachment
  □ **e.** Diabetic retinopathy
**Question 2 What clinical signs can you see?**
  □ **a.** Grey ‘red reflex’
  □ **b.** Wrinkled and folded retina
  □ **c.** Swollen optic disc margins
  □ **d.** Retinal haemorrhages and exudates
  □ **e.** The normal choroidal blood vessels cannot be seen
**Question 3 How should this patient be managed?**
  □ **a.** Oral acetazolamide 500 mg immediately
  □ **b.** Immediate referral to a retinal surgeon
  □ **c.** Posterior capsulotomy
  □ **d.** Advice to stop smoking and abstain from alcohol
  □ **e.** Routine referral for laser treatment

## ANSWERS

1) All false except d.
2) a., b. & e. are true. c. & d. are false. The disc margins are slightly blurred. This is because they are out of focus. The retina is detached, so it is in front of the disc, which means either the disc or the retina will be out of focus in fundus photos of a retinal detachment.
3) All false except b.

